# Dataset of ileum bacterial diversity in mice after heart failure due to pressure overload

**DOI:** 10.1016/j.dib.2022.108498

**Published:** 2022-07-30

**Authors:** Martina E. Spehlmann, Dhiraj P. Dhotre, Nesrin Schmiedel, Nikita Chavan, Corinna Bang, Ashraf Y. Rangrez

**Affiliations:** a Department of Internal Medicine III, Cardiology, Angiology and Intensive Care Medicine, University Hospital of Schleswig-Holstein, Rosalind-Franklin Str. 12, Kiel 24105, Germany; b National Centre for Microbial Resource, National Centre for Cell Science, Pune 411021, India; c Institute of Clinical Molecular Biology, Christian-Albrechts-University Kiel, Rosalind-Franklin-Strasse 12, Kiel 24105, Germany; d DZHK (German Centre for Cardiovascular Research), Partner Site Hamburg/Kiel/Lübeck, Germany; e Department of Internal Medicine III, University of Heidelberg, Im Neuenheimer Feld 410, Heidelberg 69120, Germany; f DZHK, Partner Site, Heidelberg, Mannheim, Germany

**Keywords:** Heart failure, Gut microbiome, Ileum, Gut-heart axis, Dysbiosis

## Abstract

We recently reported the correlation of gut bacterial diversity with heart failure using a mouse model of heart failure due to pressure overload induced by transverse aortic constriction (TAC). We found that gut the bacterial diversity is significantly altered and is directly correlated to the severity of heart failure (Heart Failure Severity Closely Correlates with Intestinal Dysbiosis and Subsequent Metabolomic Alterations (Spehlmann, 2022). In addition, stool samples that were collected for the gut microbial diversity analysis, we dissected ileum from the mice after 42 days of TAC. The total DNA was extracted to identify the bacterial diversity resided in ileum using 16S rRNA gene amplicon shotgun sequencing and downstream bioinformatics analysis to determine if it is correlated to the heart failure.

## Specifications Table

**Table:** 

Subject	Microbiology: Microbiome
Specific subject area	Host-Microbiome Crosstalk in Cardiometabolic Diseases
Type of data	Graph Figure
How the data were acquired	The ileum total DNA was extracted using QIAamp DNA stool kit, which was then used to amplify the variable regions v1-v2 of 16S rRNA gene and sequenced on an Illumina MiSeq (2 × 300 bp).
Data format	Analyzed
Description of data collection	Transverse aortic constriction (TAC) was used to induce heart failure in mice, whereas, sham operated mice were used as experimental controls. Animals were kept for 6 weeks post surgeries followed by echocardiography and tissue harvest including ileum for 16S rRNA gene amplicon shotgun sequencing.
Data source location	• Institution: University Medical Centre Kiel • City/Town/Region: Kiel • Country: Germany
Data accessibility	Sequencing data was deposited to the European Nucleotide Archive (under the accession number PRJEB45533).
Related research article	M.E. Spehlmann, A.Y. Rangrez, D.P. Dhotre, N. Schmiedel, N. Chavan, C. Bang, O.J. Muller, Y.S. Shouche, A. Franke, D. Frank, N. Frey, Heart Failure Severity Closely Correlates with Intestinal Dysbiosis and Subsequent Metabolomic Alterations. Biomedicines 10 (2022) 809. 10.3390/biomedicines10040809

## Value of the Data

• Data presented in this article indicates that heart failure does not affect ileum bacterial diversity.
• These findings will be helpful for the researchers working on gut-heart axis to focus more on stool as a reference sample.
• On a broader level, our findings are indicative of the fact that different disease manifestations are likely to affect bacterial diversity in the gut differently.

## 1 Data Description

**Fig. 1:**
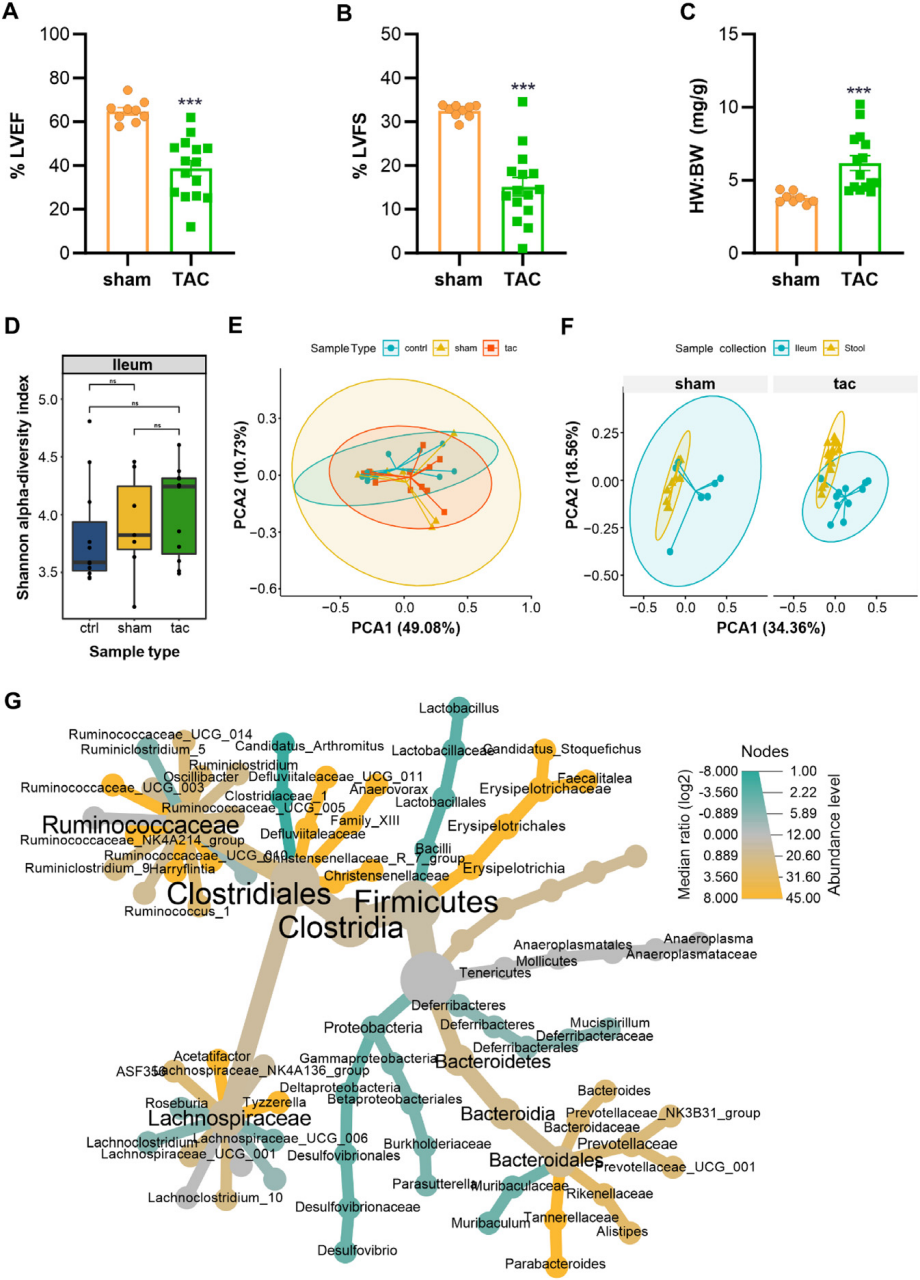
**Ileum microbial diversity in mice after 6 weeks of TAC.** Echocardiography parameters represented as bar graphs indicate ejection fraction (**A**) and fractional shortening (**B**) **C:** Bar graph showing heart weight to body weight ratios. **D:** Shannon alpha diversity calculation of different sample-types of ileum samples showing no statistically significant differences in either of the experimental groups. **E:** PCOA analysis using GuniFrac distances of different sample-types of ileum, showing no significant clustering within sample-types data. (PERMANOVA *p-value: 0.909,* Beta dispersion *p-value: 0.9066)***F:** PCOA analysis of ileum and fecal samples for sham and TAC datatypes showing significant clustering of fecal and ileum TAC samples. (PERMANOVA *p-value: 0.001,* Beta dispersion *p-value: 1e-04)* (N = Sham – 9, TAC – 15). **G:** Heat-tree of ileum Vs stool sample-type comparisons at genus level indicating most of genera belonging to Firmicutes phylum were differentially abundant in ileum/stool comparison. tac: group of mice that underwent transverse aortic constriction surgery; sham: age/gender matched mice that underwent sham surgery which followed the exact procedure as tac except for no aortic constriction; ctrl: age/gender matched mice that did not undergo any surgery.

We recently reported the correlation of gut bacterial diversity with heart failure using a mouse model of heart failure due to pressure overload induced by transverse aortic constriction (TAC). We found that gut the bacterial diversity is significantly altered and is directly correlated to the severity of heart failure (Heart Failure Severity Closely Correlates with Intestinal Dysbiosis and Subsequent Metabolomic Alterations [1]). Since ileal bacterial diversity is also affected in inflammatory bowel and related diseases, we analyzed ileal bacterial diversity to investigate if heart failure plays any role in ileal dysbiosis. Transverse aortic constriction (TAC) was used to induce heart failure in mice due to pressure overload (Fig. 1A–C). Shannon alpha diversity index to show ileum bacterial composition in sham and TAC operated mice is represented in Fig. 1D. PCoA analysis with GuniFrac distances to compare clustering of ileal bacterial diversity is shown in Fig. 1E. Further comparison of ileum against fecal bacterial diversity using PCoA analysis is shown in Fig. 1F. Differentially abundant genera between the ileum and fecal samples is compared by Wilcoxon's test (Fig. 1G).

## 2 Experimental Design, Materials and Methods

### 2.1 Animals

Male C57BL/6 J mice (6-weeks old, purchased from Charles River Laboratories, Sulzfeld, Germany) were used for the present study. Mice were acclimatized to the conditions in our animal facility for 2 weeks before start of the experiments. All mice were fed a standard rodent chow diet and kept under 12-h light and dark cycle.

### 2.2 Transverse Aortic Constriction

Mice underwent TAC (or sham) surgeries as described earlier [2]. Briefly, 8-weeks old mice were anesthetized (with buprenorphine), intubated, and kept under anesthesia (by 3% isoflurane) throughout the procedure. Body temperature was maintained by temperature regulation pads. A lateral thoracotomy through the second intercostals space was carried out to reach the transverse aorta and a suture (using Prolene 6.0) was placed around the aorta between the brachiocephalic and the left carotid artery against a 27-gauge needle. Sham group underwent same procedure as TAC without applying suture around the aorta, whereas, we used an additional age-matched control group where mice did not undergo any surgical procedure.

### 2.3 DNA Extraction and Sequencing of Bacterial 16S rDNA

DNA extraction, PCR and 16S rRNA gene sequencing was carried out as described earlier [1]. Briefly, the total DNA from fecal/ileum samples from the experimental mice was extracted using QIAamp DNA stool kit (Qiagen) on an automated platform of QIAcube. The isolated DNA was used to amplify the variable region v1-v2 of 16S rRNA gene as previously described [3]. After normalizing the PCR products on SequalPrep Normalization Plate Kit (Life Technologies), pooled samples were sequenced on an Illumina MiSeq (2 × 300 bp). The 16 s rRNA gene sequencing results were submitted to the European Nucleotide Archive (ENA) under the accession number PRJEB45533 (https://www.ebi.ac.uk/ena/browser/view/PRJEB45533?show=reads).

### 2.4 Processing of 16S rRNA Data

Analysis of the 16S rRNA gene sequences was carried out as previously described [1]. Briefly, we first used DADA2 pipeline (v1.13.1) to identify Amplicon Sequence Variants (ASVs) [4], where, raw reads were pre-processed by trimming at 240 nucleotide positions followed by filtering with a quality cut-off of 20 & an expected error threshold of 2. Filtered reads were then dereplicated, denoised, and chimeric sequences were removed. Only ASVs with an overall abundance of more than 10 were considered for taxonomic assignments using Ribosomal Database Project (RDP) naive Bayesian classifier [5] with SILVA (v132) database [6]. Further analysis of the data was carried out in R (v3.6) using phyloseq [7], vegan [8], microbiome [9], metgenomeSeq [10] and ggplot2.

### 2.5 Bacterial Compositional Analysis

Bacterial composition was analyzed as described earlier [1]. Shannon alpha diversity indices were calculated to analyze microbial diversity and richness using phyloseq R package. Whereas, the beta-diversity analyses were performed using GUniFrac and ggplot2 R packages, where, the significant grouping in samples was determined by permutational multivariate analysis of variance (PERMANOVA). Linear discriminant analysis effect size (LefSe) was used from galaxy server to identify differentially abundant ASV's within different sample types [11]. Wilcoxon test was performed to identify difference in microbial abundances in two groups, where, P-values were adjusted using false discovery rate (FDR) method.

### 2.6 Statistics

Statistical significance for bacterial composition was analyzed by non-parametric Wilcoxon's test followed by significant grouping using PERMANOVA. Whereas, the statistical significance for microbial abundances in two groups was calculated by Wilcoxon test and the P-values were adjusted using FDR. Mice echocardiography results were analyzed by one-way ANOVA (analysis of variance) followed by Student–Newman–Keuls post-hoc test. P-values ≤ 0.05 were considered statistically significant.

## Ethics Statements

All animal experiments complied with the ARRIVE guidelines and were carried out in accordance with the EU Directive 2010/63/EU for animal experiments. The ethics protocol was approved by the Ministry of Energy Transition, Agriculture, Environment, Nature and Digitalization (MELUND) of the state of Schleswig-Holstein, Germany with the reference number V241–60,965/2017 (129–10/17).

## CRediT authorship contribution statement

**Martina E. Spehlmann:** Conceptualization, Resources, Formal analysis, Writing – original draft, Writing – review & editing. **Dhiraj P. Dhotre:** Software, Formal analysis, Data curation, Supervision. **Nesrin Schmiedel:** Methodology, Formal analysis. **Nikita Chavan:** Methodology, Software, Formal analysis, Visualization. **Corinna Bang:** Methodology, Resources. **Ashraf Y. Rangrez:** Conceptualization, Writing – original draft, Writing – review & editing, Visualization, Supervision.

## Declaration of Competing Interest

The authors declare that they have no known competing financial interests or personal relationships that could have appeared to influence the work reported in this paper.

## Data Availability

Gut microbiome and metabolome of transverse aortic constriction (TAC) induced mice (Original data) (European Nucleotide Archive).
